# Impact of the “BALatrine” Intervention on Soil-Transmitted Helminth Infections in Central Java, Indonesia: A Pilot Study

**DOI:** 10.3390/tropicalmed4040141

**Published:** 2019-12-06

**Authors:** Darren J Gray, Johanna M Kurscheid, MJ Park, Budi Laksono, Dongxu Wang, Archie CA Clements, Suharyo Hadisaputro, Ross Sadler, Donald E Stewart

**Affiliations:** 1 Department of Global Health, Research School of Population Health, the Australian National University, Acton, ACT 2601, Australia; darren.gray@anu.edu.au; 2 College of Nursing, Konyang University, Deajeon 35365, Korea; mjpark.kyu95@gmail.com; 3 Yayasan Wahanna Bakti Sehatera (YWBS) Foundation, Semarang 50183, Indonesia; dokterbudilaksono@gmail.com; 4 Research School of Population Health, Australian National University, Acton, ACT 2601, Australia; dongxu.wang0714@gmail.com; 5 Faculty of Health Sciences, Curtin University, Bentley, WA 6102, Australia; archie.clements@curtin.edu.au; 6 Telethon Kids Institute, Nedlands, WA 6009, Australia; 7 Poltekkes Kemenkes Semarang 50268, Indonesia; suharyo.hadisaputro@undip.ac.id; 8 School of Medicine, Griffith University and Menzies Health Institute Queensland, Brisbane, QLD 4222, Australia; ross.sadler@griffith.edu.au (R.S.); donald.stewart@griffith.edu.au (D.E.S.)

**Keywords:** water, sanitation and hygiene (WASH), latrine intervention, soil-transmitted helminths, Indonesia

## Abstract

Many latrine campaigns in developing countries fail to be sustained because the introduced latrine is not appropriate to local socio-economic, cultural and environmental conditions, and there is an inadequate community health education component. We tested a low-cost, locally designed and constructed all-weather latrine (the “BALatrine”), together with community education promoting appropriate hygiene-related behaviour, to determine whether this integrated intervention effectively controlled soil-transmitted helminth (STH) infections. We undertook a pilot intervention study in two villages in Central Java, Indonesia. The villages were randomly allocated to either control or intervention with the intervention village receiving the BALatrine program and the control village receiving no program. STH-infection status was measured using the faecal flotation diagnostic method, before and eight months after the intervention. Over 8 months, the cumulative incidence of STH infection was significantly lower in the intervention village than in the control village: 13.4% vs. 27.5% (67/244 vs. 38/283, *p* < 0.001). The intervention was particularly effective among children: cumulative incidence 3.8% (2/53) for the intervention vs. 24.1% (13/54) for the control village (*p* < 0.001). The integrated BALatrine intervention was associated with a reduced incidence of STH infection. Following on from this pilot study, a large cluster-randomised controlled trial was commenced (ACTRN12613000523707).

## 1. Introduction

The global prevalence of infection with soil-transmitted helminths (STH) remains high, with 1.5 billion people infected worldwide, many of them children [1]. Over two thirds of STH infections are in Asia, mostly in Southeast Asia [2]. The prevalence of STH infection in Indonesia is high at 45–65%, with areas having poor sanitation reaching 80% prevalence [3]. In Central Java, research into STH infection among elementary school children by Laksono and later the Health Department, found an infection prevalence of 84–96% [4,5]. More recently, a cross-sectional survey in Semarang, Central Java, found a prevalence of 34% among a cohort of 6466 people aged between two and 93 years [6].

Anthelmintic drugs aimed at reducing morbidity are effective, but only temporarily, with a cure often followed by subsequent reinfection [7]. In rural areas, open defecation coupled with poorly constructed or inadequate latrines allows STH eggs to spread infection. Therefore, for long-term prevention, improved sanitation and community education are essential. A recent systematic review and meta-analysis concluded that “integrated control approaches emphasizing health education and environmental sanitation are needed to interrupt transmission of STH” [8]. In particular, interventions that improve the hygienic disposal of faeces to reduce soil and/or water contamination have been identified as a key strategy to control transmission and prevent related diseases [9,10,11].

In Indonesia, open defecation is common, with 55% of the poorest and 18% of the richest households practicing open defecation [12]. In 2010, less than 40% of the people in rural areas had improved latrines [13], defined as facilities that hygienically separate human excreta from human contact [14]. The country did not reach its Millennium Development Goal of 75% sanitation coverage by 2015 [15]. In rural areas, which include 118 million people, or 46.3% of the country’s population, it has been estimated that 47% of the population have improved latrines, 12% shared latrines, 12% other unimproved latrines and 29% no latrines (i.e., practice open defecation) [16]. Compounding the problems caused by the lack of improved latrines is inappropriate hygiene-related behaviour, particularly related to hand washing, with 2007 National baseline data indicating that less than a quarter (23.2%) of the population had appropriate hand-washing behaviour [17].

The aim of this study was to develop and test an integrated approach to the prevention of STH infection and reduction of both transmission and reinfection. Our intervention included anthelmintic drugs, the construction and adoption of improved latrines, and effective education regarding hygienic and sanitary behaviour.

## 2. Methods

### 2.1. Study Design

**Figure 1:**
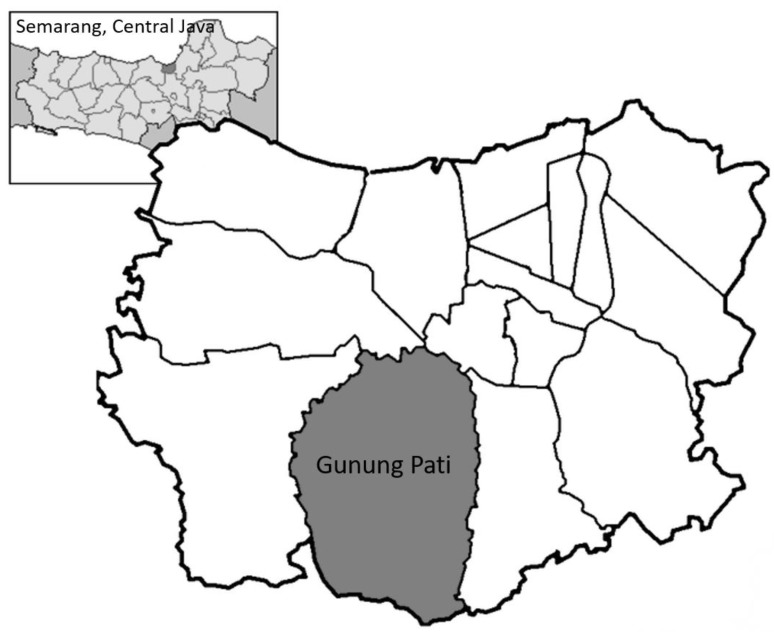
Map of Gunung Pati subdistrict in Semarang, Central Java (source: Wikipedia Indonesia, 2017).

This study was conducted in two villages, Palemon and Cepoko, in the Gunungpati sub-district of the city of Semarang, Central Java, Indonesia (see Figure 1), from July 2011 to June 2012. A random selection was made from these villages regarding which one should receive the integrated intervention and which one should be the control, by researchers who had no prior knowledge or contact with the villagers or village officials. The two villages though similar in size and local topography, were not in close proximity to each other. The study area is wooded and hilly and most of the houses, often made of local brick, are constructed by the householders themselves. More than half of the households in the study villages did not have their own latrines. A randomly selected cohort (control: n = 244; intervention: n = 283) was followed over the eight-month duration of the study. A questionnaire was administered at baseline and follow-up to all village residents, with the eligibility criterion of being over two years of age. Participants also provided two stool samples for parasitological examination. Following the baseline survey, all residents (regardless of infection status) were treated with anthelmintic medication and the incidence of STH infection was assessed at follow-up. For ethical reasons, participants who were found to be STH positive at follow-up were re-treated.

### 2.2. Ethics 

Ethical approval was given by the Semarang City authorities (ref. 070/613/IV/2011), and from the Human Research Ethics Committee at Griffith University (ref. PBH/17/11/HREC).

### 2.3. Procedure

**Figure 2:**
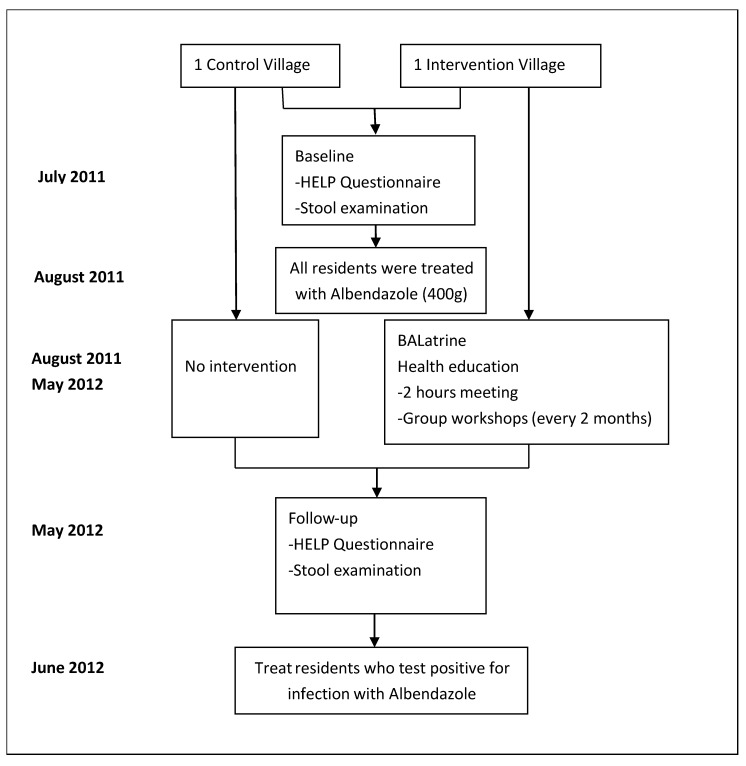
Flowchart of the study.

Our study procedure reflected the integrated model previously described (see also Figure 2), comprising chemotherapy, a locally constructed latrine (the “BALatrine”) and community health education. Following WHO Guidelines, a single oral dose of Albendazole (400mg) was administered immediately after the baseline survey [1].

**Figure 3:**
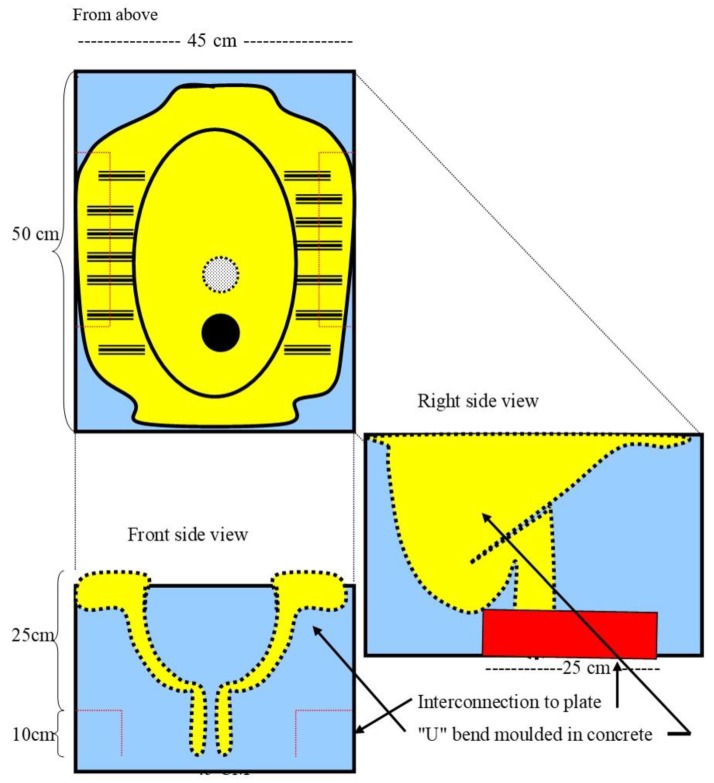
Schematic presentation of the BALatrine.

The BALatrine is designed for resource poor rural communities and emergency situations, to be made by local people using local materials. Testing for cultural acceptance was conducted in the field through pilot studies in Pekalongan in Central Java and BALatrines were also used in an emergency refugee camp during the 2010 eruption of the Mt Merapi volcano, where they were proven appropriate for the level of technology available in the village context [18,19]. The BALatrine is a relatively simple squat latrine (Figure 3) that can be constructed by village residents using inexpensive materials. When water for flushing is available, a U-bend (‘goose-neck’) water closet can be attached. When water is not available, such as during the dry season, the latrine can be used in a dry-pit configuration (with removal of the U-bend attachment), with a lid to isolate it from insects and to prevent odours from escaping [20]. Thus, it can function despite seasonal changes in water supply. Besides being inexpensive (cost at the time of the study was $50 USD for the latrine and local building materials; equating to approximately $80–90 at the time of publication), for people with limited financial and educational resources it is easy to copy. Its construction and use reflect critical resource, environmental and technical issues and due to local village input it also overcomes some major disincentives embodied in conventional latrines by being culturally familiar, simple and easy to use.

The community health education programme is an essential adjunct to latrine construction. In the intervention village, all residents were given health education regarding hygiene, sanitation, and prevention of STH infections. This health education component was delivered via community meetings in each village. All village residents were invited and meetings were held in the village meeting hall. The health education/health promotion component of the intervention was implemented through a two-hour village-wide mobilization meeting, which formed the project launch and was designed to mobilize households by consciousness-raising and provision of information about parasite infection and burden of STH. Subsequently, a series of small group workshops took place with the villagers in order to describe the BALatrine construction in detail and how to plan, construct, use, and maintain their latrines, as well as to discuss STH disease pathways. The content of the health education programme comprised information about the dangers of STH infections and, using illustrated leaflets, how the transmission of STH infections can be prevented by the construction of latrines and with appropriate hygiene-related behaviours.

### 2.4. Measurements and Analyses

The primary outcome measures of the integrated intervention were STH infection status at baseline and at follow-up. STH infection status was measured through laboratory analysis of stool samples collected from each participant at baseline and at follow-up eight months after the BALatrines were constructed. The samples were analysed microscopically for the presence of helminth eggs, according to the Willis-Mollay Flotation technique [21]. After preparing the samples, a cover slip was placed over each sample tube and left for 10 minutes. After 10 minutes, a drop of eosin solution (2%) was added to a glass slide onto which the cover slip was then placed and observed using a light microscope at 10 × magnification. A positive sample was where at least one STH egg was identified.

A face-to-face questionnaire (the Helminth Education and Latrine Project (HELP) questionnaire) was also administered at baseline and follow-up and this provided information about villagers’ demographic attributes. We also assessed local village environmental contamination with faeces. These findings have been published elsewhere [22,23].

Data were analysed using SPSS Version 22 (IBM, New York, United States), Microsoft Excel and the tools at Open Source Epidemiologic Statistics for Public Health [24]. Differences between participants in control and intervention villages were analysed using the unpaired t-test and the Pearson’s chi-squared (χ^2^) test. Logistic regression analysis was performed and odds ratios calculated. To compare the intervention village with the control village, we report both crude odds ratios and adjusted for age and sex.

## 3. Results 

### 3.1. Characteristics of the Participants

There were 527 participants in the study at baseline, 244 in the control village and 283 in the intervention village. Their ages ranged from three to 70 years, with a mean ± SD of 29.4 ± 16.1 years in the control village and 32.2 ± 17.9 years in the intervention village (Table 1; for the age difference between villages, *p* = 0.06).

In both villages, similar proportions of the participants had completed elementary education (control, 211/244, 86.5%; intervention, 253/283, 89.4%). In the control village, 98.8% of the residents had a monthly income below 1 M Indonesian Rupiah (IDR) or about US$70, whereas in the intervention village the comparable percentage was 97.2% (*p* = 0.017). In the control village, 38.5% of the residents lived in a home in which all floor spaces were dry, whereas in the intervention village the comparable percentage was 54.1% (*p* < 0.001). In the intervention village greater than 90% of households adopted the BALatrine, as measured at the follow up.

### 3.2. STH-Infection Status

At baseline, the prevalence of STH infection was almost the same in the two villages (Table 1). At follow-up, the cumulative incidence of infection was much lower in the intervention village than in the control village (Table 2, 13.4% vs. 27.5%, *p* < 0.001). After adjustments for age and gender, the benefit of the intervention was still clear (adjusted odds ratio = 0.38, 95% CI 0.25–0.60, Table 2). The intervention was particularly effective in children (adjusted odds ratio = 0.12, 95% CI 0.03–0.56, Table 2).

## 4. Discussion

Improving access to adequate sanitation is a critical step toward the sustainable interruption of STH transmission. Yet this is an ongoing challenge in low resource settings where public sewerage system infrastructure rarely exists, on-site sanitation systems are either improperly designed or poorly functioning, and open defecation is seen as culturally acceptable, especially in rural areas [25,26]. Often people must rely on shared sanitation facilities, which have been shown to increase the risk of adverse health outcomes compared to individual household latrines [27]. Compounding the issue is limited access to materials and a lack of technical expertise to build or improve latrines [26,28].

The BALatrine was specifically designed to overcome many of these challenges whilst also taking into consideration cultural appropriateness and acceptability. Using simple technology and locally sourced, inexpensive materials, the BALatrine is cheap, easy to build and maintain and adaptable for wet and dry conditions. Building the latrines through community mobilisation also helps keep the costs down and enables the householders themselves to take ownership over the latrines and their maintenance and consequently, benefit from improved health, helping alleviate the cycle of poverty that is often associated with STH infections [26].

In the present study, we evaluated the effectiveness of an integrated BALatrine intervention at reducing human worm burden through a pilot study in two villages in Central Java. People in the intervention village were 2.6 times less likely to be infected following the BALatrine-based intervention than those in the control village indicating that the BALatrine is associated with a reduced worm burden. However, it is important to note that we did not resurvey participants after baseline deworming to determine the efficacy of the Albendazole treatment nor did we differentiate between STH species, which are known to respond differently to treatment [29,30]. We have based our interpretation of the results on the assumption that treatment was effective at temporarily reducing infections to zero. Consequently, it is possible that the effect seen could be a result of differential cure rates between villages based on their STH profile at baseline. However, a prevalence study we conducted the following year (manuscript under review) across 16 villages in two neighbouring subdistricts of Semarang, including Gunung Pati, revealed *Ascaris lumbricoides* as the predominant species (mean prevalence of 26% vs. 7.9% and 1.8% for hookworm and *Trichuris trichuria*, respectively). We therefore believe that it is not unrealistic to assume that our study villages also had similar burdens of each of the STH species at baseline and that treatment would be similarly effective across the two sites.

Our study also found that nearly all households adopted the BALatrine suggesting a strong willingness among villagers to improve sanitation and a desire to improve the health of their families. Access to improved latrines does not guarantee their use, however, particularly over time when old habits or cultural preferences can be difficult to overcome [25,28,31]. Integrating health education and promotion programmes to improve peoples’ understanding and knowledge of the link between open defecation and ill-health and WASH-related behaviours, is therefore extremely important [25,28]. People also need to understand the importance of properly cleaning and maintaining their latrines, particularly as this is associated with higher latrine use [28]. In the present study, a community health education programme was administered prior to the construction and installation of the latrines and aimed to raise awareness, improve hygiene behaviours and motivate the villagers to build and continue to use their new latrines, which may have led to the high uptake observed in this study. However, we did not assess the impact of this program on participants’ knowledge and behaviour change or assess latrine use over time, which are key limitations of this study.

It is well established that chemotherapy alone will not break the transmission cycle. Recent studies have also shown that programmes solely focusing on WASH have limited effect on STH incidence and may provide no additional impact compared to mass drug administration programmes alone [32,33,34,35]. In contrast, health education and promotion programmes can be highly effective at reducing the incidence of STH infections if designed appropriately such as the highly successful “Magic Glasses” study, which resulted in a 50% reduction in STH infections [36]. However, sustained reinforcement of health messages is required in order to increase their effectiveness over the longer term [37,38]. Ultimately, eliminating STH will be best achieved through integrated control programmes. The current study adds to the growing body of research into the impact of integrated control programs on soil-transmitted helminthiases and demonstrates that sanitation interventions can be effective at reducing worm burden when designed appropriately for the local context and combined with health education and promotion.

In conclusion, our findings provide “proof of principle” that the BALatrine-based intervention is effective in preventing STH infection. We will now undertake a full-scale randomized controlled trial and contribute much needed evidence based on WASH and STH.

## Figures and Tables

**Table 1:** Baseline characteristics of participants.

Village Status	Control	Intervention
Village	Cepoko	Palemon
Sample Size	244	283
Mean Age (years)	29.4	32.2
Prevalence of STH infection: % (95% CI)	21.7% (16.5–26.9)	25.8% (20.7–30.9)
Sex Ratio (F/M)	141/103	151/132
Prevalence of STH infection by Sex (F/M)	22.0%/21.4%	20.5%/31.8%

**Table 2:** Infection rates in the control and intervention villages.

Variable	Control	Intervention	Odds Ratio		Odds Ratio	
			Crude	*p* Value	Adjusted *	*p* Value
**All participants**	**n = 244**	**n = 283**				
Prevalence of infection at baseline: % (95% CI)	21.7 (16.5–26.9)	25.8 (20.7–30.9)	-	-	-	-
Cumulative incidence of infection at follow-up: % (95% CI)	27.5 (21.9–33.1)	13.4 (9.5–17.4)	0.41 (0.26–0.64)	<0.001	0.38 (0.25–0.60)	<0.001
**Children**	**n = 54**	**n = 53**				
Prevalence of infection at baseline: % (95% CI)	18.8 (8.2–28.9)	18.9 (8.3–29.4)	-	-	-	-
Cumulative incidence of infection at follow-up: % (95% CI)	24.1 (12.7–35.5)	3.8 (0.0–8.9)	0.12 (0.03–0.58)	0.01	0.12 (0.03–0.56)	0.01

* The model for all participants was adjusted for age and sex. The model for children (<14 years) was adjusted for gender only.
